# Understanding implementation context and social processes through integrating Normalization Process Theory (NPT) and the Consolidated Framework for Implementation Research (CFIR)

**DOI:** 10.1186/s43058-022-00264-8

**Published:** 2022-02-09

**Authors:** Dawn Schroeder, Thea Luig, Tracy L. Finch, Sanjay Beesoon, Denise Lynn Campbell-Scherer

**Affiliations:** 1 Physician Learning Program, University of Alberta Faculty of Medicine & Dentistry, Edmonton, Alberta Canada; 2 Department of Nursing, Midwifery & Health, Northumbria University, Newcastle upon Tyne, UK; 3 Surgery Strategic Clinical Network, Alberta Health Services, Edmonton, Alberta Canada; 4 Office of Lifelong Learning & Physician Learning Program, University of Alberta Faculty of Medicine & Dentistry, Edmonton, Alberta Canada; 5 Department of Family Medicine, Faculty of Medicine & Dentistry, University of Alberta, Edmonton, Alberta Canada; 6 Alberta Diabetes Institute, University of Alberta, Edmonton, Alberta Canada

**Keywords:** Normalization Process Theory, NPT, Consolidated framework for implementation research, CFIR, Theories, Frameworks, Qualitative, Methodology, Implementation process, Implementation context

## Abstract

**Background:**

For successful implementation of an innovation within a complex adaptive system, we need to understand the ways that implementation processes and their contexts shape each other. To do this, we need to explore the work people do to make sense of an innovation and integrate it into their workflow and the contextual elements that impact implementation. Combining Normalization Process Theory (NPT) with the Consolidated Framework for Implementation Research (CFIR) offers an approach to achieve this. NPT is an implementation process theory that explains how changes in the way people think about and use an innovation occurs, while CFIR is a framework that categorizes and describes contextual determinants across five domains that influence implementation. We demonstrate through a case example from our prior research how we integrated NPT and CFIR to inform the development of the interview guide, coding manual, and analysis of the findings.

**Methods:**

In collaboration with our stakeholders, we selected NPT and CFIR to study the implementation process and co-developed an interview guide to elicit responses that would illuminate concepts from both. We conducted, audio-recorded, and transcribed 28 interviews with various professionals involved with the implementation. Based on independent coding of select transcripts and team discussion comparing, clarifying, and crystallizing codes, we developed a coding manual integrating CFIR and NPT constructs. We applied the integrated codes to all interview transcripts.

**Results:**

Our findings highlight how integrating CFIR domains with NPT mechanisms adds explanatory strength to the analysis of implementation processes, with particular implications for practical strategies to facilitate implementation. Multiple coding across both theoretical frames captured the entanglement of process and context. Integrating NPT and CFIR enriched understandings of how interactions between implementation processes and contextual determinants shaped each other during implementation.

**Conclusion:**

The integration of NPT and CFIR provides guidance to identify and explore complex entangled interactions between agents, processes, and contextual conditions within and beyond organizations to embed innovations into routine practices. Nuanced understandings gained through this approach moves understandings beyond descriptions of determinants to explain how change occurs or not during implementation. Mechanism-based explanations illuminate concrete practical strategies to support implementation.

**Supplementary Information:**

The online version contains supplementary material available at 10.1186/s43058-022-00264-8.

Contributions to the literature

- This article provides a theory-based methodological process to investigate the entanglement of implementation processes and contextual determinants through the integration of NPT and CFIR at all stages of the research process.
- NPT and CFIR are complementary in that CFIR provides constructs to describe contextual determinants and NPT helps us to understand through theoretical explanations the processes that shape those determinants.
- Making visible the interactions of implementation processes and contextual factors to support individuals practicing or studying improvement or implementation efforts.

## Background

For successful implementation of an innovation within a complex adaptive system, we need to understand the ways that implementation processes and their contexts shape each other [1]. To do this, we need to understand the work people do to make sense of an innovation and integrate it into their workflow and the contextual elements that impact implementation. The cognitive and social work to achieve this is conceptualized in May and Finch’s Normalization Process Theory (NPT). NPT is a prominent social process theory that provides the mechanisms to explain how and why the cognitive and social processes of individuals and collectives within their context are critical for implementation [2]. Damschroder and colleagues’ Consolidated Framework for Implementation Research (CFIR) is a determinant framework that draws from several theories to categorize and describe contextual factors across five domains [3]. CFIR’s comprehensive selection of determinants within these domains acts as an anchor to support a systematic approach to think through and explore determinants relevant to an implementation project. Both NPT and CFIR are widely used on their own in implementation projects [4–10]. A small number of studies and study protocols, including our prior qualitative study on the implementation of the National Surgical Quality Improvement Program (NSQIP), applied both NPT and CFIR to inform different aspects of an implementation study [11–16]. Although the purpose of NPT and CFIR are different, they are complementary. CFIR offers a taxonomy of static qualities of determinants to consider at multiple levels within and beyond the organization whereas NPT characterizes the mechanisms to explain why and how change occurs to support new practices [2, 3]. Using NPT and CFIR together to guide an implementation project provides a theoretical grounding to gain rich insights into the emergent and shifting interplays between the work people do and the context in which they are situated to implement an innovation.

Currently, there is a gap in the literature in relation to *how* and *why* the integration of NPT and CFIR as a combined analytical framework reveals a broad and nuanced understanding of implementation work. To contribute to the implementation science literature on how and why the integration of these two approaches is of value, the objectives for this paper are (1) to illuminate through a case example from our prior research [11] the value in integrating NPT with CFIR to guide interview guide development, coding, and further analysis of data; and (2) to make transparent and accessible how, methodologically, NPT and CFIR can be used together in a rigorous way. We begin with a brief overview of our case example [11] before we use select exemplars to discuss why and how designing an implementation study using NPT and CFIR in combination revealed nuanced understandings of implementation work and context. Lastly, we discuss key methodological insights derived from our experience integrating NPT with CFIR. We follow the Standards for Reporting Qualitative Research (Additional file 1. Standards for Reporting Qualitative Research) [17].

## Methods

### Case example: National Surgical Quality Improvement Program (NSQIP) [11]

The aim of NSQIP is to improve the quality and safety of surgical care [18]. NSQIP is a widely used comprehensive data platform that enables hospitals to continuously track and measure risk-adjusted surgical morbidity and mortality outcome data to inform quality improvements (QI) [19]. This data is used by clinicians and healthcare leaders to identify areas where QI is needed as well as monitor the impact of QI changes. The provincial Surgery Strategic Clinical Network (Surgery SCN), Alberta Health Services approached the Physician Learning Program (PLP) at the University of Alberta to conduct a qualitative exploratory study to capture and explain how the work people did to implement NSQIP interacted with contextual conditions to integrate or not the innovation across five hospital sites. The PLP creates clinically actionable information to address clinical gaps, including information on implementation, and co-creates sustainable interventions to advance practice [20].

### Theoretical rational for selecting and integrating CFIR and NPT

Birken and colleagues’ [21] usability, applicability, and acceptability criteria were considered in our selection of a relevant framework, model, and/or theory to guide the implementation case example study [11]. CFIR’s comprehensive listing of constructs relevant to the healthcare sector at multiple conceptual levels (e.g., individual, team, organization, and beyond) and the familiarity of its constructs to stakeholders considering factors and questions they wished to explore met the usability and acceptability criteria. However, given that determinant frameworks do not address how change occurs [22], NPT was chosen as another theoretical lens to meet the usability criteria because it aligned with our aim of understanding the mechanisms to explain how change occurs during implementation. Finally, the applicability for use in qualitative implementation research was met for both approaches.

### Data collection methods, recruitment, and analysis

Using our NPT-CFIR integrated qualitative study design, we developed an interview guide based on constructs from both approaches (Additional file 2 Semi-Structured Interview Guide with NPT and CFIR Cross-Referencing). We used purposive and snowball sampling to recruit participants and conducted individual interviews with healthcare professionals (*N*=28) within and outside of the implementation teams across several hospital sites. Implementation teams consisted of Surgical and Anesthesia Physician Champions, Surgical Clinical Reviewers (a nursing role responsible for collecting and inputting surgical data and facilitating QI efforts driven by NSQIP data), and Directors/Managers of surgical services. Inductive and deductive coding based on NPT and CFIR constructs was applied to code transcript data and develop the coding manual. Further thematic analysis of the data revealed how NPT and CFIR constructs worked together to understand how contextual conditions and the work people did to implement the innovation shaped each other. We used NVIVO V. 12 (QRS International (Americas)) to manage the data. Methods and substantive findings for the NSQIP study are described elsewhere [11]. Ethics approval for the case example study was provided by the Health Research Ethics Board at the University of Alberta (Pro00088327), and operations’ approval was granted by Alberta Health Services [11].

### Consolidated Framework for Implementation Research

Damschroder and colleagues’ Consolidated Framework for Implementation Research (CFIR) is a comprehensive determinant framework that lists and describes 39 constructs synthesized from a review of theories that propose determinants believed to influence implementation of an innovation (Additional file 3: Definitions for the Consolidated Framework for Implementation Research Domains and Constructs). The constructs are categorized within five general domains to represent multiple conceptual levels [3]. The intervention characteristics domain informs exploration of determinants such as perception of an innovation’s *adaptability* to meet an organization’s needs or the *relative advantage* of using an innovation over existing ways of doing things. Constructs within the inner setting domain point to determinants such as *networks and communications* to assess the quality of the connections between individuals and groups, and *compatibility* between the goals of leadership and the meanings others attach to the intent of implementing the intervention. Constructs in the outer setting domain direct attention to the extent to which *external policies* and/or *cosmopolitanism*, defined as information sharing among individuals and/or groups outside of the target site, may influence implementation. The process domain describes activities relevant to implementation—such as “*planning, engaging, executing, and reflecting and evaluating*” and key roles such as *champions* and *opinion leaders*. Lastly, the individual characteristics domain includes individual perception of *self-efficacy* in using an innovation and individual *knowledge and beliefs.* In summary, CFIR describes static qualities of an innovation, context, and individuals at a given point in time that may influence implementation efforts [3]. Paying attention to these qualities during the planning and execution phases of a change in practice and knowing the state of such qualities as determinants of successful implementation may help identify contexts where implementation may be more or less successful. Yet, this knowledge does not help us understand how change occurs individually, collectively, and organizationally to achieve these states [22]. Combining NPT with CFIR helps to capture and explain how dynamic implementation processes interact with static qualities to change the way people think about, utilize, and integrate an innovation into routine practices.

### Normalization Process Theory

The aim of May and Finch’s Normalization Process Theory (NPT) is to understand and explain the cognitive and social processes people engage in to do the work to integrate and sustain the use of an innovation in everyday practices [2]. NPT is an extension of May and colleagues’ Normalization Process Model (NPM) [23]. NPM was developed through secondary analyses of healthcare studies to identify empirical generalizations to explain the *collective actions* people engage in to routinize innovations. To understand and explain how these collective actions are shaped, they later expanded on NPM to develop a formal theory that includes three additional generative mechanisms—*coherence*, *cognitive participation*, and *reflexive monitoring* [24].

NPT is used to explore qualitatively and/or quantitatively *what* the work is to implement new technologies into everyday practice settings and explain *how* the work is done through four non-linear core generative mechanisms—Coherence, Cognitive Participation, Collective Action, and Reflexive Monitoring. Each core mechanism has four sub-components to guide inquiries (Additional file 4: Definitions for Normalization Process Theory Core Mechanisms and Sub-Components). We drew on these mechanisms to explore: (1) the sense-making individuals and groups engage in to develop an understanding of how an innovation is different from other practices, the benefits in using it and what people need to do to use it (Coherence); (2) relational work to ensure the right people are involved with implementing the innovation, and legitimation and commitment among groups of people to re-organize themselves to use it (Cognitive Participation); (3) efforts of people to operationalize an innovation (Collective Action); and (4) individual and collective appraisal of the work to implement and sustain its use (Reflexive Monitoring) [2]. Combining NPT with CFIR provides complementary lenses to understand complex dynamic interplays between contextual conditions and social and cognitive processes that shape context.

## Results

In this section, we describe how we applied NPT and CFIR constructs at each stage of the research process and provide exemplars from our case example [11] to illuminate how these approaches integrate and complement each other. A summary of steps and key considerations to take into account when applying NPT and CFIR to study implementation are provided in Table 1. Additional select examples to illuminate how NPT and CFIR constructs interacted to inform nuanced understandings of the implementation work are provided in Table 2. It is important to note that in table 2, CFIR constructs were selected based on their relevance for the case study and that the areas of integration between CFIR and NPT highlighted in the table emerged from our data [11].

**Table 1:** A guide to applying NPT and CFIR to understand interactions between processes and context in the evaluation of implementation work

Stage	Key considerations
1. Ensuring methodological fit	• Ensure implementation project and evaluation/research aim fit with scope and focus of NPT and CFIR • Consider using a theory and framework criteria matching tool (such as T-Cast [21]) • Engage with stakeholders for contextual understanding and to develop shared understandings of methodological fit between project objectives and the integrated use of NPT and CFIR
2. Developing the interview guide	• Open-ended questions to explore bi-directional connections between contextual conditions and the work people do to implement an innovation • Prompt interviewee to recall specific situations • Cross-referencing each question with prompts as memory aids to further explore NPT and CFIR constructs • In-depth familiarity with NPT and CFIR constructs is key to rapidly respond with prompts to explore constructs as they emerge in interviews and to inquire about constructs that are absent • Regular discussions with stakeholders and pilot testing ensure comprehensiveness and applicability
3. Developing the coding manual	• Initial inductive coding allows for exploration of aspects of an implementation experience not anticipated from NPT and CFIR constructs • Integration of NPT and CFIR constructs and inductive codes based on regular meetings among research team members to reach a consensus on the coding structure • Multiple coding highlights where CFIR and NPT integrate and complement each other
4. Analyzing the qualitative data	• Openness to explore the complexities through applying multiple codes to interview passages • Using multiple coding to think through a web of connections across CFIR domains and NPT mechanisms to move beyond description to explain how and why change may occur to integrate an innovation into routine practices
5. Reporting the findings	• Clearly describe the methodology of integrating NPT and CFIR in the methods • When describing results focus on the interaction and entanglement, rather than separating results by processes and contextual factors • Reflect on and report pros and cons of applying NPT and CFIR together in your study

**Table 2:** Areas of interaction, overlap, and difference among select CFIR constructs with NPT mechanisms and sub-concepts

CFIR constructs	NPT constructs	Interaction of CFIR and NPT	Select case examples
***Intervention characteristics***			
Evidence, strength and quality	Coherence	The sensemaking work people did to see value in using the innovation and come to a shared understanding with others on the benefit of the innovation involved a perception and appraisal of the strength and quality of the evidence.	One physician champion who was keen to bring NSQIP to his site referred to the program as the “alpha and omega of pooled data in the world”.
Relative advantage	Coherence	The perception of a relative advantage involved sensemaking to understand how the innovation differed from previous practices to understand the value and benefits over existing ways of working.	A director making sense of the advantage in using NSQIP to identify areas to focus on Quality Improvement (QI) found the continuous tracking of data through NSQIP was better than previous ways of identifying issues for QI, stating: “it would’ve been manual tracking…if we wanted to take a look at say blood clots or infections…operationally we would like to do that but just sometimes don’t have the time to do it”.
Adaptability	Collective action	The way the innovation can be shaped to different contexts may impact on all aspects of collective action.	An implementation team modified the way data was presented by aggregating data for each surgical specialty. This action mitigated feelings of being individually targeted and promoted positive relational integration.
***Outer setting***			
Patient needs and resources	Coherence and collective action	The sensemaking work people did in using the innovation to identify gaps in patient needs through the NSQIP data led implementation team members to think about relationship building with healthcare providers outside of the surgery unit to address patient needs and resources post-discharge from the hospital.	Sensemaking through the 30-day follow up phone call to patients (a component of NSQIP) and NSQIP data outside of the surgical area (such as emergency room visit data) highlighted a need to improve patient discharge teaching. A QI Consultant stated: “Patients are coming into our emergency department and not understanding their wound and what they needed to do with it or what to look for…led to the identification of a need for more “education and information sharing with patients on many different levels”.
Cosmopolitanism	Coherence and cognitive participation	Cosmopolitanism points to the key influence of context outside the organization for implementation, a domain that is outside the scope of NPT. Sensemaking work to understand one’s role in using an innovation and how to use it relied largely on the connection and exchange with external provincial implementation teams.	The data quote below points to the critical role of experienced surgical clinical reviewers outside of the expansion hospital sites in helping new implementation team members understand how to use NSQIP. A Surgical Clinical Reviewer stated: “So we were able to get quick contact with the SCRs, over on the pilot site hospitals, and just to see how they do things, how they organise their days, what their priorities are, how they balance work demands, and kind of how they saw their program roll out.” [11]
***Inner setting***			
Networks and communications	Coherence, cognitive participation, and collective actions.	Sensemaking to understand role commitments outside of implementation work and ways to overcome time or workload constraints led to collective actions to strengthen communication channels among newly formed implementation teams.	At one site, efforts were made to meet in unconventional spaces to keep each other informed of NSQIP work and to solve challenges as they occurred. This work led to a strong sense of teamness that positively contributed to implementation efforts. SCR stated: “whether it’s email, text, popping up to the O.R. between cases, they are open to meeting with me and are very responsive in that way" [11].
Culture	Coherence and cognitive participation	Sensemaking work among individuals and teams required a better understanding of values and norms across different professional groups involved. This understanding supported the engagement with professional groups to help with sensemaking around what the innovation is and its value. This work led to legitimation within and between professions to integrate the innovation into routine practices.	After being denied a presence at certain physician meetings, an SCR spent several months building relationships with physicians by helping them to see what his/her role was with NSQIP and how to interpret and use the data. This work led to the re-organizing of relationships as physicians started to see value in the SCR role and NSQIP. The SCR stated: “Now they’re coming to me almost as like a general problem-solver like, Oh what do you think about this or Maybe can you do this project”.
Implementation climate includes six sub-constructs: *Compatibility; relative priority; organizational incentives and rewards; goals and feedback; and learning climate*	Coherence, cognitive participation, and collective action	The sensemaking work people engaged in to understand initial reactions to an innovation and the work they did to help others see value in and legitimation for being involved with the innovation led to an alignment between meanings and values leadership and professional groups attached to the innovation (compatibility) and led to improvements in relational integration for the innovation.	The examples below illustrate incompatibility issues between leadership and physicians on the meanings attached to NSQIP. The sensemaking work implementation teams engaged in to understand why some physicians felt threatened by the program led to relational work and thoughtful messaging to help physicians see value in using the program and to reassure them of the intent of using the data. Operating Room Manager: “the perception at the beginning of it was, they’re looking at my quality, right, like how am I doing and they’re going to use my infection, you know…But (SCR Name) seemed to kind of persevere and, you know, continue to articulate the methodology, to help them understand better what we’re using the information for”. One Physician Champion involved in a meeting to understand physician concerns about NSQIP learned about their fears such as “the data being left out and missing the curve, you know your numbers are showing you’re not doing a good job [11] and individualized compared to the other guys…you have to spend some time reassuring them”. One Physician Champion used deliberate language to present NSQIP data as a tool to use as a collective to inform QI initiatives to try to avoid misrepresentations of the data as a way to individually monitor a surgeon’s work: “The whole point of collecting this (referring to data), is so that we, global we, institution, can get better at what we do…I just stress the objectivity of it…These are the numbers that we’re getting. I‘m not pointing the finger at any particular individual or any particular group of individuals or any particular practice.” [11]
Readiness for implementation includes three sub-constructs: Leadership engagement; available resources; and access to information and knowledge	Coherence and cognitive participation	Leadership engagement involved sensemaking work among Directors and Managers to identify and connect new implementation team roles with existing groups to support integration of the innovation. Availability of resources to support implementation of an innovation can positively or negatively influence sensemaking and legitimation work among individuals and/or groups.	Director: “I participate in the surgeon meetings, add input into kind of priority setting with her (referring the SCR), kind of help influence some of those initiatives in the other units, for her (referring to the SCR), when she gets pushback from leadership around that” [11] SCR at a different site: “It was very helpful in that she (Manager) quickly incorporated us into our larger team… So our executive directors, all her direct reports…And then also through those connections with the other managers, they’ve invited us to their quality council meetings and that kind of stuff. So that’s beneficial”. In this example, the lack of material resources to support sensemaking for one Physician put them at risk of not seeing value in contributing to the work (legitimation): “My problem is that a lot of these meetings are on Skype for Business, and I don’t have access to that. So, I don’t have a computer where I could see any of the PowerPoint presentations. So, I just have to dial in to the audio and figure it out…So for me it’s hard…I’m losing interest as a result because, you know in my hospital doctors don’t have computers” [11].
***Characteristics of individuals***			
Knowledge and beliefs about the intervention	Coherence and cognitive participation	Engaging in sensemaking work to understand what an innovation is about and how it can be used in current practices can positively or negatively change prior knowledge and beliefs and result in the reorganizing or not of relationships to integrate it into practice.	Initially, a physician champion was excited about using NSQIP data to inform QI work. As they learned more about the data they could collect and the data they needed, they realized the innovation was not a good fit. This resulted in low cognitive participation efforts to move implementation forward. “What was first perceived as a powerful tool became a very vanilla bland look at some variables” [11]
Self-efficacy	Coherence, cognitive participation, and collective action	Belief in one’s capabilities to do the work required sensemaking work to understand what the implementation role required in relation to knowledge and skills. This work preceded actions to address deficits in collective action’s skill set workability to confidently perform an implementation role.	Several SCRs did not have QI work experience before assuming the implementation role. QI learning needs were supported by leadership through enrolment in QI courses at the start of their implementation role and connections between SCRs and QI consultants. Connections between SCRs and QI Consultants led to newly formed working relationships in QI informed by NSQIP data.
***Process***			
Planning	Coherence and cognitive participation	Implementation planning interacts in a cyclical way with individual and collective sensemaking work (coherence) and cognitive participation. It is key for planning to build a shared understanding of the goals and the value of the innovation and to work together to engage key actors to build a community of practice around the innovation. This work informs understandings of how to perform new tasks, what actions, procedures, roles and processes are needed to integrate the work and the innovation into existing workflows.	Creating time for understanding what NSQIP can do to inform improvement in surgical care and how to do the work of NSQIP data collection and of building a community of practice around NSQIP was key to implementation planning. Engagement of leadership and implementation teams within each site and across the province was crucial to help SCRs make sense of their work, how to set up processes, and how to connect to the different professional groups and key actors they needed to regularly interact with to drive NSQIP forward.
Engaging	Coherence, cognitive participation, and collective action	Engaging is related to cognitive participation as both describe the relational work to embed and sustain a new practice into existing workflows. Coherence work intersects with enrolling people in participating and in understanding what their tasks and contributions may be. CFIR highlights the role of a champion, NPT helps understand how a champion is key to helping others with sensemaking (coherence) and recognizing the value in being involved with the innovation. This complex relational work informs collective action that brings people together in new ways (relational and contextual integration) to work with the innovation.	In this example, the physician champion discussed their understanding of what was behind hesitancies among some physicians about NSQIP. Their narrative reveals the work they did to engage physicians and help them understand the NSQIP data. It also illuminates the leadership style they used to provide guidance and facilitate physician engagement while preserving autonomy as physician groups reorganized themselves to work with data from NSQIP. Physician Champion: “They were worried, with the implementation, that people were going to feel that this was monitoring and that there was going to be punitive issues. I am aware of how we disseminate… actually helping people interpret, and that’s where we’re at…I’m trying to tailor it so that each group has their opportunity to deal with it, however they want. So as an example, general surgery…they have a robust well attended monthly business meeting, where they will discuss…trends…But they’ll also talk about upticks in things like infection rates. They may not, again, come up with initiatives about how to address it, but at least it’s part of what they do”.
Reflecting and evaluating	Reflexive monitoring	This CFIR construct refers to using quantitative and qualitative feedback in an ongoing reflection and evaluation of implementation progress and experiences. NPT sub-constructs of reflexive monitoring explore in more detail the individual, collective appraisal of the worth of the new practice, and a systemization of a variety of information, including quantitative and qualitative data, to appraise and potentially redefine and modify the implementation process, or even aspects of the practice.	This example illuminates an individual’s experience appraising the impact of NSQIP implementation work on their clinical and leadership work. Physician champion: “If I was going to do it I don’t think…needs two champions. I think (hospital name) needs maybe one champion that has to take a more, give themselves more time. Because that’s my biggest problem…I wouldn’t make it a Chief of Surgery or Anesthesiology, I would try and find a third person because we’ve got so many other things.”

### Integrating NPT and CFIR to develop the interview guide and data collection

Applying constructs from a process theory and a determinant framework to guide each stage of the research process provided a systematic approach to identify conditions that influence implementation and to explore how change occurred in the way people think about and use an innovation. We used broad open-ended questions to elicit and explore the implementation experience the interviewee may have had while capturing the entangling of social processes and contextual determinants. Question 2 in the interview guide (Additional file 2: Semi-Structured Interview Guide with NPT and CFIR Cross-Referencing)—*Can you walk me through how NSQIP was put into place at your hospital?*—is an example of one of the open-ended questions that asks the participants to recall aspects of a specific situation that they may otherwise consider obvious or irrelevant but may be vital to answering the research question. To ensure relevant constructs within a participant’s responses are explored, it is imperative that the interviewer become deeply familiar with the constructs before data collection begins. It is through this familiarity that rapid connections between what is being talked about with relevant constructs can be made to prompt the participant to explore related experiences more deeply or to explore constructs that are absent in the conversation. To support the interviewer, several prompting questions were attached to open-ended questions to act as memory aids to delve more deeply into relevant constructs. For example, asking prompt 2c—*what type of training did you receive?*—opened the conversation to formal and informal training experiences that relate to NPT and CFIR constructs. One response to question 2c was as follows:

They (Leadership) really encouraged me to do the AIW course and the Prosci course and just different things to really broaden my… knowledge… different things that you want to build in the team when you’re doing change management. Because that was such a new area to me. (SCR #617)

In the quote above, the respondent raised aspects of their training that relate to NPT’s *coherence* and CFIR’s *cosmopolitanism*, *leadership engagement*, and *self-efficacy constructs.*

Another example is Question 9—*How has use of the NSQIP tool changed your relationships with other surgical staff and with management?* which was designed to illuminate constructs such as NPT *coherence*, *cognitive participation*, and *collective action* constructs and/or CFIR *inner setting* constructs. The following extract illustrates part of a response that spoke to *coherence* and *collective action*.

I think, in the past, it would have been, if we had something like a mortality, morbidity round, we would say it was just for a given group of surgeons and now, I think, we're more likely to have anaesthesia available for all discussions, as opposed to ones that just pertained to that area”... “And then I'd say, similarly, the areas of activity in the hospital, I think, again, we're starting to bring groups together. (Physician Champion #120)

In the above quote, an implementation team member reflected on how bringing different groups of professionals together changed the way these groups interact with each other and how they made sense of the work on QI initiatives driven by the NSQIP data. Constructs within this passage relate to CFIR’s *inner setting’s networks and communications* and NPT’s *coherence* and *collective action’s relational integration*.

### Integrating NPT and CFIR to guide coding and analysis of the findings

Coding is a vital first step in understanding where and how constructs from determinant frameworks and process theories integrate. Inductive coding allows for exploration of aspects of an implementation experience not anticipated from constructs [25] before deductively coding data as it relates to the constructs. In the coding process, rigor was strengthened through triangulation by having regular meetings between researchers involved in the process to discuss and reach a consensus on the codes to develop a coding manual [26].

We assigned multiple codes to each interview passage that represented multiple aspects of the complex issues that arose in response to interview questions. Multiple coding highlighted where CFIR and NPT integrate and complement each other. What was important was to keep intact the connections between the static qualities and implementation processes to inform further analysis. Using this approach revealed the entanglement of multiple processes and determinants within the implementation experience. Next, we provide select examples to show how NPT and CFIR integrated.

Within the *intervention characteristics domain*, we found that NPT’s *differentiation* construct related to CFIR’s *relative advantage* construct when the data pointed to peoples’ perceptions of differences between an innovation and current practices. A key distinction is that NPT’s *differentiation* gets at the work people do individually and collectively to understand how an innovation is different from the usual way of doing things in order to assess whether or not a *relative advantage* exists. Other CFIR constructs within this domain, such as *trialability*, *adaptability*, and *cost*, complemented NPT by providing descriptions of intervention qualities to explore, which are not defined within the NPT constructs. However, CFIR constructs such as *adaptability* is linked to actions people take and their experiences in getting things to work which is conceptualized within NPT’s *collective action* domain.

CFIR’s *outer setting domain* provides constructs to describe the context outside of an implementation target organization, which again is not in the scope of NPT. However, NPT mechanisms may enhance the understanding of how determinants in the *outer setting* facilitate implementation. For example, the importance of *cosmopolitanism* as a CFIR construct in the *outer setting* was better understood through our data that showed how the sharing of information and experiences between implementation teams among several organizational sites created a crucial space to do *coherence* and *legitimation* work to support sensemaking and a community of practice. In our data, relating *coherence* and *legitimation* work to *cosmopolitanism* provided insights on how provincially supported monthly conferences for site champions and surgical clinical reviewers created a learning space to develop shared understandings about the value in working with NSQIP (*coherence*) and to learn through the experiences of others different ways to move forward with implementation work. For example, participants at several sites recalled how helpful the monthly conferences were in thinking through how to interpret the data and use it to achieve quality improvement goals without overwhelming colleagues with unrealistic workloads. In this example, the regular connection to other teams outside of the organization (*cosmopolitanism*) was crucial to build relationships to support sensemaking (*coherence*) and the belief that it is right to be involved with NSQIP work (*legitimation*). Other CFIR constructs within this domain provide descriptions of possible external influences, such as *external policies and incentives*, *peer pressure*, and *patient needs and resources*.

In CFIR’s *inner setting domain*, the determinants within the *structural characteristics*, *networks and communications*, *culture*, *implementation climate*, and *readiness for implementation* constructs describe and speak to complex dynamic conditions that interact with each other to influence implementation. In our data, we found CFIR’s *compatibility* as part of the *implementation climate* construct related to NPT’s *relational integration*, *legitimation*, and *coherence* work*.* However, the purposes of these constructs are clearly different. While CFIR describes *compatibility* as a fit between knowledge and meanings attached to an innovation by the users and those in decision-making roles involved in the adoption of an innovation, NPT constructs relate to the work people do to build and sustain support for and confidence in an innovation’s usefulness in everyday practices [2, 3]. Relating *compatibility* to NPT processes such as *coherence*, *legitimation*, and *collective action* aided our understanding of how implementation processes and context shape each other. For example, actions taken by implementation teams, such as holding public forums for physicians to communicate their concerns about the innovation, informed initial understandings of where the resistance to the intervention was coming from. This sensemaking work identified perceptions among some surgeons that data sampling would potentially miss data with no surgical complications and therefore lead to erroneous conclusions about a surgeon’s performance. To mitigate these concerns, some implementation teams deliberately framed the data as “our data” to work with to improve surgical care as a collective and presented the results as aggregate surgical specialties. This change in communication was part of the *legitimation* and *relational integration* work and resulted in positive changes within the *implementation climate* to support better and more productive engagement among physicians and within the implementation teams.

Within CFIR’s *process domain*, *planning*, *engaging*, *executing*, and *reflecting and evaluating* are described as four essential implementation processes. Descriptions within these constructs highlight the importance of developing a plan guided by a change theory to support change at the individual and organizational levels, engaging key roles known to influence uptake of an innovation, assessing quality of executing the implementation processes, and learning what implementation efforts worked well and what strategies need to change through reflection and evaluation. To design tangible supports for implementation processes, we need an understanding of the work needed to develop a good plan. Whereas CFIR describes key roles, such as *opinion leaders*, *champions*, and *external change agents*, that are influential in engaging others to see value in using an innovation, NPT guides us to explore the work people in these roles do to drive change in how others value or make sense of an innovation.

In our data, we found the overlapping of CFIR’s *engaging* construct with NPT constructs, such as *coherence*, *cognitive participation*, and *collective action* gave more granularity to guide the analysis as we asked what, who, and how the work was done to successfully or not integrate the innovation into existing workflows. As a result, we integrated all four NPT mechanisms and sub-constructs as nodes in the CIFR *process domain* in the coding manual as they provided the granularity to capture the work needed to *engage*, *execute*, *and reflect.*

Lastly, CFIR’s *individual characteristics domain* describes several individual qualities that may influence change at the individual and/or organizational level. We found in our data that CFIR’s individual *self-efficacy* and *knowledge and beliefs* constructs related to NPT’s *coherence*, *enrolment*, and *interactional workability* constructs. It was these work processes that elicited changes in perceptions about the innovation itself and beliefs in one’s capacity to carry out the work occurred.

In all five CFIR domains, NPT identified the processes to negotiate and shape the contextual determinants influencing implementation efforts while CFIR provided additional constructs outside the scope of NPT to consider at each step of the research process. The following two data examples further demonstrate the entangling of determinants and processes to illuminate how NPT and CFIR constructs worked well together in the analysis phase.

Our first example relates to an interview passage that we coded with CFIR’s *compatibility* and *external change agent* constructs and NPT’s *coherence* and *cognitive participation* constructs. In this passage, a presentation from an *external change agent* (NSQIP Provincial Lead) to introduce NSQIP to a group of physicians at an implementation site was an action that targeted *coherence* work. The disparity between the meanings attached to the intervention by leadership in the *outer setting* and the physician group within this new implementation site resulted in resistance. Using NPT and CFIR constructs to probe more deeply during the interview and to guide the coding and analysis helped to explore how initial resistance among several physicians was addressed by the implementation team as a problem of *compatibility* of the innovation by engaging with physicians and teams in *coherence* and *legitimation* work. The relational work that ensued to help others come to a shared understanding (*coherence*) of the intent of the innovation helped physicians see value (*legitimation*) in using it to improve surgical quality of care. This led to the restructuring of relationships where implementation team members and physicians worked collaboratively with the program data to identify areas to work on quality improvement initiatives and co-create solutions. From this example, it is clear that without the *coherence* and *legitimation* work to help others see value in using an innovation, it is difficult to *enroll* individuals to help move implementation efforts forward.

Our second example relates to an interview passage that we coded with CFIR’s *external change agent*, *cosmopolitanism*, and NPT’s *coherence* and *cognitive participation* constructs. Within the *process domain*, the *engaging* construct describes an *external change agent* as a role that formally facilitates implementation from outside of an organization. NPT constructs gave more granularity of what the work was of an *external change agent* (the NSQIP Provincial Lead) to support implementation teams through the building of a community of implementation teams across the province to share and translate knowledge about the innovation (*cosmopolitanism*). The monthly conference call was one strategy used by the *external change agent* to support and shape ongoing *coherence* and *legitimation* among implementation team members across the province. This support was critical to the relational restructuring that needed to occur within teams to support implementation efforts for if team members do not see value in a program and/or believe that they should be involved, the development of a committed team is at risk for failure. Additionally, the creation of these communities of practice with other organizations was a platform for experienced and new implementation team members along with the *external change agent* to share and learn through their experiences different ways to create awareness for and integrate the innovation into existing workflows as a tool to drive quality improvement efforts.

## Discussion

Our experience combining NPT with CFIR to guide the NSQIP study led to three key methodological and theoretically informative insights. Our first insight relates to the synergies between these approaches, where the NPT propositions provided more granularity to understand the work to engage others and integrate an innovation into existing work flows. NPT focuses on how the cognitive and social processes of individuals and teams within their structural context interact with and can change determinants in the *individual characteristics*, *inner* and *outer setting*, and *process domains.* Furthermore, NPT’s generative mechanisms—*coherence*, *cognitive participation*, and *reflexive monitoring*—provide granularity to the invisible elements behind individual and collective actions of those involved with implementation. A recent review of work combining realist approaches and NPT in implementation studies similarly found that NPT provided additional explanatory power for better understanding implementation [27]. Whereas CFIR provides key determinants to consider within the *outer setting* and *intervention characteristics* domains, which are outside of the scope for NPT.

Our second insight is practical, in the sense that integration of constructs applied to data containing entangled processes and determinants support the aim to not reduce and simplify complexity in order to understand how processes and determinants work together. CFIR-based questions and prompts provide a systematic approach to identify determinants at multiple conceptual levels while NPT-based questions and prompts help to capture crucial data to illuminate the mechanisms that foster or discourage actions to integrate and routinize an innovation. Maintaining these connections at each step of the research project made visible how and why contextual determinants and processes shape each other to provide comprehensive explanations of implementation work. During analysis, multiple coding of narrative excerpts captures the non-linearity of social processes and contextual determinants within implementation work. Applying multiple frameworks to study implementation has been described as complicated and yet valuable [16]. In our view, the value in using an implementation theory and a determinant framework to explore the complexities within the implementation landscape is compelling because it permits a more granular understanding of the phenomenon. Maintaining complexity through the integration of NPT and CFIR to capture the entanglement of processes and contextual determinants was a methodological strength in our approach. Rich insights gained through this integrative approach outweighed additional time taken in the beginning to combine NPT and CFIR to develop the interview guide, coding structure, and further guide the analysis.

Finally, our third insight relates to how using these two approaches illuminate concrete, practical strategies for stakeholders to support their ongoing implementation efforts, such as providing time for people to engage individually and collectively in sensemaking work. Additionally, our work contributes context-specific granularity to literature on implementation strategies [28, 29] by illuminating through a qualitative analytical approach how barriers, such as not having enough time to engage in sensemaking impact implementation understandings and actions at an individual and collective level.

In sociology, Carl May [30] further evolved NPT to propose that all implementation processes are embedded in complex social systems that shape agency and therefore need to be understood as dynamic social processes that form and are molded by contextual elements. Our methodological approach aligns with this argument by showing how and why the integration of a mid-range theory and a determinant framework lens takes us beyond the describing of static enablers and barriers to capture nuanced explanations [31] of the dynamic evolving relationship between agency, social processes, and contextual conditions. According to Greenhalgh and Papoutsi [32], efforts to spread innovations across multiple complex organizational settings often benefit from the combining of different perspectives—such as social science, complexity science, and/or implementation science—to understand how and why implementation efforts fail or succeed. Drawing on all three as interrelated and complementary approaches recognizes the complex adaptive nature of healthcare work and organizations involving people with their individually and collectively negotiated norms and practices and is key to achieving the practical results of moving evidence into practice to improve the quality and safety of care.

### Limitations

In this paper, explanations for the intersections between all NPT and CFIR constructs in the data is not exhaustive due to time limitations for participants to engage in an interview. Absence of data examples to illuminate other constructs, such as cost or adoption, was due to their relevance at a higher governance level than that of the participating sites and individuals in the case example study. For future research, it would be beneficial to systematically study the integration between NPT and all 39 CFIR constructs.

## Conclusion

Combining CFIR and NPT consistently and rigorously throughout research design, data collection, and analysis is one option to achieving an in-depth understanding of how context constrains agency and agency shapes constraints. The synergistic use of NPT onto CFIR provides guidance to explore the complex interactions of processes and contextual conditions within and beyond organizations and the local work needed to routinize innovations into existing workflows. Nuanced understandings and mechanism-based explanations gained through the combining of these approaches support organizational leaders in their efforts to develop implementation strategies that support individuals and groups working to change practices within their unique contexts.

## Supplementary Information

**Additional file 1.** Reporting checklist SRQRR1.

**Additional file 2.** Semi-structured interview guide.

**Additional file 3.** Definitions for CFIR domains & constructs.

**Additional file 4.** Definitions for NPT.

## Data Availability

The data sets used and/or analyzed during the current study are available from the corresponding author upon reasonable request.

## Abbreviations

NPT: Normalization Process Theory
CFIR: Consolidated Framework for Implementation Research
NSQIP: National Surgical Quality Improvement Program
SCR: Surgical Clinical Reviewer
QI: Quality Improvement

## References

[1] May C, Johnson M, Finch T (2016). Implementation, context and complexity. Implement Sci.

[2] May C, Finch T (2009). Implementing, embedding, and integrating practices: an outline of normalization process theory. Sociology..

[3] Damschroder LJ, Aron DC, Keith RE, Kirsh SR, Alexander JA, Lowery J (2009). Fostering implementation of health services research findings into practice: a consolidated framework for advancing implementation science. Implement Sci.

[4] Drew S, Judge A, May C, Farmer A, Cooper C, Javaid MK (2015). Gooberman-Hill and the REFReSH study group. Implementation of secondary fracture prevention services after hip fracture: a qualitative study using extended normalization process theory. Implement Sci.

[5] Hooker L, Small R, Humphreys C, Hegarty K, Taft A (2015). Applying normalization process theory to understand implementation of a family violence screening and care model in maternal and child health nursing practice: a mixed method process evaluation of a randomised controlled trial. Implement Sci.

[6] Atkins S, Lewin S, Ringsberg KC, Thorson A. Provider experiences of the implementation of a new tuberculosis treatment programme: a qualitative study using the normalization process model. BMC Health Serv Res. 2011;11:275. 10.1186/1472-6963-11-275 PMC3215962 22004533

[7] Nordmark S, Zingmark K, Lindberg I. Process evaluation of discharge planning implementation in healthcare using normalization process theory. BMC Med Inform Decis Mak. 2016;16:48. 10.1186/s12911-016-0285-4 PMC4847180 27121500

[8] Soi C, Gimbel S, Chilundo B, Muchanga V, Matsinhe L, Sherr K (2018). Human papillomavirus vaccine delivery in Mozambique: identification of implementation performance drivers using the consolidated framework for implementation research (CFIR). Implement Sci.

[9] Stokes T, Tumilty E, Doolan-Noble F, Gauld R (2018). Health pathways implementation in a New Zealand health region: a qualitative study using the consolidated framework for implementation research. BMJ Open.

[10] Damschroder LJ, Lowery JCL (2013). Evaluation of a large-scale weight management program using the consolidated framework for implementation research (CFIR). Implement Sci.

[11] Schroeder D, Luig T, Beesoon S, Robert J, Campbell-Scherer D, Brindle M. What work is required to implement and sustain the national surgical quality improvement program (NSQIP)? A qualitative study of NSQIP implementation in Alberta, Canada. BMJ Open. 2021;11. 10.1136/bmjopen-2020-044720. 10.1136/bmjopen-2020-044720 PMC8483041 34588226

[12] Desveaux L, Beauchamp MK, Lee A, Ivers N, Goldstein R, Brooks D. Effects of a community-based, post-rehabilitation exercise program in COPD: protocol for a randomized controlled trial with embedded process evaluation. JMIR Res Protoc. 2016;5(2):e63. 10.2196/resprot.5435 PMC4880740 27169436

[13] Scalia P, Durand MA, Forcino RC, Schubbe D, Barr PJ, O’Brien N, et al. Implementation of the uterine fibroids option grid patient decision aids across five organizational settings: a randomized stepped-wedge study protocol. Implement Sci. 2019;14:88. 10.1186/s13012-019-0933-z PMC6721118 31477140

[14] Kairy D, Messier F, Zidarov D, Ahmed S, Poissant L, Rushton PW, et al. Evaluating the implementation process of a new telerehabilitation modality in three rehabilitation settings using the normalization process theory: study protocol. Int J Healthc Manag. 2019;12(4):348–55.

[15] Carbonneau M, Eboreime EA, Hyde A, Campbell-Scherer D, Faris P, Gramlich L, et al. The cirrhosis care Alberta (CCAB) protocol: implementing an evidence-based best practice order set for the management of liver cirrhosis - a hybrid type 1 effectiveness-implementation trial. BMC Health Serv Res. 2020;20:558. 10.1186/s12913-020-05427-8 PMC7301349 32552833

[16] Connell LA, McMahon NE, Harris JE, Watkins CL, Eng JJ. A formative evaluation of the implementation of an upper limb stroke rehabilitation intervention in clinical practice: a qualitative interview study. Implement Sci. 2014;9:90. 10.1186/s13012-014-0090-3 PMC4156624 25112430

[17] O’Brien BC, Harris IB, Beckman TJ (2014). Standards for reporting qualitative research: a synthesis of recommendations. Acad Med.

[18] Beesoon S, Sydora BC, Thanh NX, Chakravorty D, Robert J, Wasylak T (2020). Does the introduction of American College of Surgeons NSQIP improve outcomes? A systematic review of the academic literature. J Am Coll Surg.

[19] ACS National Surgical Quality Improvement Program. Accessed 10 Mar 2021. https://www.facs.org/Quality-Programs/ACS-NSQIP

[20] Physician Learning Program. Accessed 21 June 2021. https://www.albertaplp.ca/

[21] Birken SA, Rohweder CL, Powell BJ, Shea CM, Scott J, Leeman J (2018). T-Cast: an implementation theory comparison and selection tool. Implement Sci.

[22] Nilsen P. Making sense of implementation theories, models and frameworks. Implement Sci. 2015;10:53. 10.1186/s13012-015-0242-0 PMC4406164 25895742

[23] May C, Finch T, Mair F, Ballini L, Dowrick C, Eccles M (2007). Understanding the implementation of complex interventions in health care: the normalization process model. BMC Health Serv Res.

[24] May C, Mair F, Finch T, MacFarlane A, Dowrick C, Treweek S (2009). Development of a theory of implementation and integration: Normalization Process Theory. Implement Sci.

[25] Braun V, Clarke V (2006). Using thematic analysis in psychology. Qual Res Psychol.

[26] Morse JM, Barrett M, Mayan M, Olson K, Spiers J (2002). Verification strategies for establishing reliability and validity in qualitative research. Int J Qual Methods.

[27] Dalkin SM, Hardwick RJL, Haighton CA, Finch TL (2021). Combining realist approaches and normalization process theory to understand implementation: a systematic review. Implement Sci Comm.

[28] Waltz TJ, Powell BJ, Fernandez ME, Aadie B, Damschroder LJ (2019). Choosing implementation strategies to address contextual barriers: diversity in recommendations and future directions. Implement Sci.

[29] Powell BJ, Waltz TJ, Chinman MJ, Damschroder LJ, Smith JL, Matthieu MM (2015). A refined compilation of implementation strategies: results from the Expert Recommendations for Implementing Change (ERIC) project. Implement Sci.

[30] May C, Finch T, Rapley T. Normalization process theory. In: Nilsen P, Birken SA, editors. Handbook on Implementation Science. Cheltenham: Edward Elgar Publishing Limited; 2020. p. 144–67.

[31] Kislov R, Pope C, Martin GP, Wilson PM (2019). Harnessing the power of theorizing in implementation science. Implement Sci.

[32] Greenhalgh T, Papoutsi C (2019). Spreading and scaling up innovation and improvement. BMJ..

