# Supplementary material for: Understanding implementation context and social processes through integrating Normalization Process Theory (NPT) and the Consolidated Framework for Implementation Research (CFIR)
Source: Implement Sci Commun. 2022 Feb 9;3:13. doi: 10.1186/s43058-022-00264-8 (PMC8826671; doi:10.1186/s43058-022-00264-8)
Supplement: Supplementary file 1 — Additional file 1. Reporting checklist SRQRR1. [file 43058_2022_264_MOESM1_ESM.docx]

**Additional file 1. Standards for Reporting Qualitative Research (SRQR)***

**Title and abstract Page/line no(s)**

| **Title** - Concise description of the nature and topic of the study identifying the study as qualitative or indicating the approach (e.g., ethnography, grounded theory) or data collection methods (e.g., interview, focus group) is recommended | P1  Lines 2 to 4 |
| --- | --- |
| **Abstract** - Summary of key elements of the study using the abstract format of the intended publication; typically includes background, purpose, methods, results, and conclusions | P3  Lines 1 to 22  and  P4  Lines 1 to 13 |

# Introduction

| **Problem formulation** - Description and significance of the problem/phenomenon studied; review of relevant theory and empirical work; problem statement | P6  Lines 1 to 23 |
| --- | --- |
| **Purpose or research question** - Purpose of the study and specific objectives or questions | P7  Lines 1 to 8 |

# Methods

| **Qualitative approach and research paradigm** - Qualitative approach (e.g. ethnography, grounded theory, case study, phenomenology, narrative research) and guiding theory if appropriate; identifying the research paradigm (e.g. postpositivist, constructivist/interpretivist) is also recommended; rationale | P8  Lines 4 to 18 |
| --- | --- |
| **Researcher characteristics and reflexivity** - Researchers’ characteristics that may influence the research, including personal attributes, qualifications/experience, relationship with participants, assumptions, and/or presuppositions; potential or actual interaction between researchers’ characteristics and the research questions, approach, methods, results, and/or transferability | P 7  Lines 20 to 23  P8  Lines 1 to 3  See Reference # 11 in the Reference List for case example used in this paper. Reference # 11 provides details on researcher characteristics. |
| **Context** - Setting/site and salient contextual factors; rationale | P8  Lines 18 to 22  P9  Line 1 |
| **Sampling strategy** - How and why research participants, documents, or events were selected; criteria for deciding when no further sampling was necessary (e.g., sampling saturation); rationale | P8  Lines 18 to 20 |
| **Ethical issues pertaining to human subjects** - Documentation of approval by an appropriate ethics review board and participant consent, or explanation for lack thereof; other confidentiality and data security issues | P9  Lines 7 to 9 |
| **Data collection methods** - Types of data collected; details of data collection procedures including (as appropriate) start and stop dates of data collection and analysis, iterative process, triangulation of sources/methods, and modification of procedures in response to evolving study findings; rationale | P8  Lines 18 to 20 |
| **Data collection instruments and technologies** - Description of instruments (e.g., interview guides, questionnaires) and devices (e.g., audio recorders) used for data collection; if/how the instrument(s) changed over the course of the study | Additional File 2. Semi-Structured Interview Guide with NPT and CFIR cross-referencing is in a separate file. |
| **Units of study** - Number and relevant characteristics of participants, documents, or events included in the study; level of participation (could be reported in results) | P8  Lines 18 to 22 |
| **Data processing** - Methods for processing data prior to and during analysis, including transcription, data entry, data management and security, verification of data integrity, data coding, and anonymization/de-identification of excerpts | P9  Lines 5 and 6 |
| **Data analysis** - Process by which inferences, themes, etc., were identified and developed, including the researchers involved in data analysis; usually references a specific paradigm or approach; rationale | P9  Lines 1 to 5  P 14  Lines 5 to 16 |
| **Techniques to enhance trustworthiness** - Techniques to enhance trustworthiness and credibility of data analysis (e.g., member checking, audit trail, triangulation); rationale | P 14  Lines 8 to 15 |

| **Limitations** - Trustworthiness and limitations of findings | P22  Lines 4 to 10 |
| --- | --- |

# Results/findings

| **Synthesis and interpretation** - Main findings (e.g., interpretations, inferences, and themes); might include development of a theory or model, or integration with prior research or theory | P 12  Lines 6 to 23  Pages 13 to 18  P19  Lines 1 to 19 |
| --- | --- |
| **Links to empirical data** - Evidence (e.g., quotes, field notes, text excerpts, photographs) to substantiate analytic findings | P13  Lines 3 to 6  Lines 14 to 19  Table 2 is indicated on page 11 line 5 and is found at the end of the manuscript file. |

**Discussion/Other**

| **Conflicts of interest** - Potential sources of influence or perceived influence on study conduct and conclusions; how these were managed | P24  Lines 11 and 12 |
| --- | --- |
| **Funding** - Sources of funding and other support; role of funders in data collection, interpretation, and reporting | P23  Lines 18 to 20  P24  Lines 1 and 2 |
| **Integration with prior work, implications, transferability, and contributions(s) to the field** - Short summary of main findings; explanation of how findings and conclusions connect to, support, elaborate on, or challenge conclusions of earlier scholarship; discussion of scope of application/generalizability; identification of unique contributions(s) to scholarship in a discipline or field | P19  Lines 20 to 23  P20 and P21  P22  Lines 1 to 3 |

*The aims of the SRQR is to provide transparency in all aspects of qualitative research by providing guidelines for reporting qualitative research

Reference:

1. O’Brien BC, Harris IB, Beckman TJ, Reed DA, & Cook DA. Standards for reporting

qualitative research: a synthesis of recommendations. *Academic Medicine.*

2014;89(9). DOI:10.1097/ACM.0000000000000388.
