# Supplementary material for: Understanding implementation context and social processes through integrating Normalization Process Theory (NPT) and the Consolidated Framework for Implementation Research (CFIR)
Source: Implement Sci Commun. 2022 Feb 9;3:13. doi: 10.1186/s43058-022-00264-8 (PMC8826671; doi:10.1186/s43058-022-00264-8)
Supplement: Supplementary file 2 — Additional file 2. Semi-structured interview guide. [file 43058_2022_264_MOESM2_ESM.docx]

**Additional file 2.** Semi-Structured Interview Guide with NPT and CFIR Cross-Referencing

| **NSQIP Semi-Structured Interview Questions** | **Corresponding NPT Constructs and CFIR Domains** |
| --- | --- |
| 1. **How did you first hear about NSQIP?**     1. What did you think of NSQIP when you first heard about it?    2. How was the decision made to adopt and make it work in your setting? How did you participate in those decisions?    3. What did you like and not like about the idea of using NSQIP? | **NPT:** Coherence, Cognitive Participation and Reflexive Monitoring  **CFIR:** Intervention characteristics and Inner setting domains |
| 1. **Can you walk me through how NSQIP was put into place at your hospital?**     1. i.e. **what happened first?** – meeting/memo?    2. Was there a plan for implementation? Were the goals and objectives clearly communicated? Who was involved in the planning?    3. What type of training did you receive?    4. Were MD Champions appointed or did they volunteer for their role?    5. Who did you go to with questions/issues, resource availability?    6. Where did you run into CHALLENGES with the NSQIP work?    7. Were other QI initiatives running at the same time – if yes, how was NSQIP impacted?    8. How did you become involved and what did you need to do to understand your role in NSQIP and make it work?    9. How did management support NSQIP? What supports did you receive from management?    10. What would you do differently if you could start over again? | **NPT:** Coherence, Cognitive Participation, Collective Action, and Reflexive Monitoring  **CFIR**: Outer and Inner setting and Process domains |
| 1. **How has NSQIP impacted your practice?**     1. How is NSQIP different from your hospital/unit’s usual ways of evaluating and identifying areas for QI?    2. How did NSQIP fit in with other priorities and daily work? How did you juggle it all?    3. Is NSQIP a normal part of your work? | **NPT:** Coherence and Collective Action  **CFIR**: Intervention characteristics, Inner setting and Process domains |
| 1. **Do you see value in using NSQIP for your work?**     1. If not in the present, what about in the future? | **NPT:** Coherence and Reflexive Monitoring  **CFIR:** Intervention characteristics, Individual characteristics and Process domains |
| 1. **What type of feedback did you receive about how implementation of NSQIP was going and how did you receive that feedback?** | **NPT:** Reflexive Monitoring  **CFIR**: Inner setting and Process domains |
| 1. **How did you meet other people who work with NSQIP both in your own hospital, across the other sites, and with the surgery SCN?**   a. How do you feel communication is going across sites and with the SCN?  b. How useful do you find this  communication for the work at  your hospital? | **NPT:** Cognitive Participation and Collective Action  **CFIR**: Inner and Outer setting domains |
| 1. **Can you describe how you and your colleagues communicate about anything related to NSQIP?**     1. If the team meets to de-brief – how often and how did these meetings go? – prompt for hallway/coffee break conversations    2. How was the team able to adapt NSQIP according to feedback that was received?    3. Ask how they communicate to each other about news, accomplishments, staff changes, problems, monthly meetings etc… | **NPT:** Collective Action and Reflexive Monitoring  **CFIR**: Inner setting, Individual characteristics and Process domains |
| 1. **What is your perception of how people in your unit/hospital felt about implementing NSQIP – at the beginning and now?**     1. Do you think there is a shared understanding among the professional groups and leadership about the purpose of NSQIP and its value? | **NPT:** Coherence  **CFIR:** Inner setting and Characteristics of individuals domain |
| 1. **How has use of the NSQIP tool changed your relationships with other surgical staff and with management?**     1. Have you noticed a difference in the relationships between colleagues and between colleagues and management since NSQIP was put into place? | **NPT:** Cognitive Participation and Collective Action  **CFIR**: Inner setting domain |
| 1. **When your hospital receives a quarterly activity report on NSQIP data, could you walk me through the process for what happens if data shows there is a need for improvement?**      - 1. Is there a QI structure/framework your team would like to use?   2. Who is the data shared with? How do you feel about the current data sharing process?   3. Who is involved with the decision-making process when choosing a QI initiative based on NSQIP data?   4. Are you aware of the NSQIP intervention care bundles? What do you think of these care bundles? | **NPT:** Coherence, Collective Action and Reflexive Monitoring  **CFIR**: Intervention characteristics and Process domains |
| 1. **What advice would you give future sites regarding implementation of NSQIP?** | **NPT:** Reflexive Monitoring  **CFIR**: Process domain |
| 1. **Is there anything else you would like to add about your experience with NSQIP?** | **Not applicable** |
