# Supplementary material for: Understanding implementation context and social processes through integrating Normalization Process Theory (NPT) and the Consolidated Framework for Implementation Research (CFIR)
Source: Implement Sci Commun. 2022 Feb 9;3:13. doi: 10.1186/s43058-022-00264-8 (PMC8826671; doi:10.1186/s43058-022-00264-8)
Supplement: Supplementary file 3 — Additional file 3. Definitions for CFIR domains & constructs. [file 43058_2022_264_MOESM3_ESM.docx]

**Additional file 3.** Definitions for the Consolidated Framework for Implementation Research Domains and Constructs.

| **Intervention characteristics domain**  ***Intervention*** ***Source*** - Perceptions regarding whether the decision to adopt an innovation was internally or externally perceived as a good idea to alleviate a problem.  ***Evidence strength and quality*** – perceptions of evidence to support an innovation.  ***Relative advantage*** of using a new innovation over existing practices.  ***Adaptability*** of an innovation to align with site needs.  ***Trialability*** of an innovation – i.e. ability to pilot a new innovation to build familiarity.  ***Complexity –*** perceived difficulties in using a new innovation.  ***Design quality and packaging*** – perceptions associated with the quality of an innovation – how it is built and presented.  ***Cost*** - associated with purchasing and implementing a new innovation. |
| --- |
| **Outer setting domain**  ***Patient needs and resources -*** Knowing patient needs as well as factors that facilitate or impede efforts to meet their needs.  ***Cosmopolitanism –*** networking and sharing of information between and outside of an organization.  ***Peer pressure –*** pressure to implement an innovation from peers at other sites.  ***External policies and incentives –*** external strategies to direct implementation efforts – i.e. external policies, regulations or guidelines. |
| **Inner setting domain**  ***Structural characteristics –*** includes size and age of an organization, administrative intensity, and level of decision-making autonomy.  ***Networks and communications –*** refers to the quality of social networks – sense of being part of a team among individuals and/or units and ways of communicating – i.e. formal or informal.  ***Culture –*** socially constructed values and norms within professions and/or of an organization.  ***Implementation climate*** – the following six sub-constructs attributed to an innovation impact the capacity and will of the organization for change: ***tension* *for* *change****;* ***compatibility*** with existing workflows and an alignment between the values and meanings individuals attach to an innovation; ***relative*** ***priority*** individuals attach to an innovation; ***organizational incentives and rewards****;* clear communication of ***goals and feedback****;* and a ***learning climate*** where leaders and team members support each other in the change process through the exchange of knowledge related to the innovation and the provision of time and space to reflect on the innovation.  ***Readiness for implementation –*** indicators of organizational commitment demonstrated by the following three sub-constructs: ***leadership engagement*** refers to the level of involvement and commitment demonstrated by managers and leaders with implementation; level of ***available resources*** to carry out the implementation – including training, education, time, physical space and money; and the ease of ***access to*** ***information and knowledge*** to support the work of those using an innovation such as: experienced staff, experts, documents and information systems. |
| **Characteristics of individuals domain**  ***Knowledge and beliefs about the innovation –*** attitudes and values individuals attach to an innovation.  ***Self-efficacy –*** perception of one’s capabilities to make necessary changes to implement an innovation.  ***Individual stage of change –*** depending on the stage of change model used, the phase an individual is in can indicate where they are in terms of use and mastery of an innovation.  ***Individual identification with organization –*** level of commitment to an organization can impact the level of effort given by individuals to the implementation of an innovation.  ***Other personal attributes*** – includes other personal traits such as: innovativeness, learning style, motivation, values, competence etc… |
| **Process domain**  ***Planning –*** the degree to which an implementation plan is developed and the degree to which it considers inputs from stakeholder perspectives, channels of communication, and strategies designed for different subgroups involved with the innovation.  ***Engaging –*** refers to the involvement of Opinion Leaders, Champions, Implementation Leaders, and External Change Agents to influence and support efforts to use an innovation. ***Executing –*** involves carrying out and evaluating the execution of a plan to understand factors such as the degree of involvement among key stakeholders, the ability to complete tasks within specified timelines and/or the quality or depth of the implementation process.  ***Reflecting and evaluating –*** qualitative and/or quantitative evaluation of implementation efforts to ascertain where improvements are needed. |

The definitions are adapted from: Damschroder LJ, Aron DC, Rosalind EK, Kirsh SR, Alexander JA, Lowery JC. Fostering implementation of health services research findings into practice: a consolidated framework for advancing implementation science. *Implement Sci.* 2009;4:50.
