# Supplementary material for: Understanding implementation context and social processes through integrating Normalization Process Theory (NPT) and the Consolidated Framework for Implementation Research (CFIR)
Source: Implement Sci Commun. 2022 Feb 9;3:13. doi: 10.1186/s43058-022-00264-8 (PMC8826671; doi:10.1186/s43058-022-00264-8)
Supplement: Supplementary file 4 — Additional file 4. Definitions for NPT. [file 43058_2022_264_MOESM4_ESM.docx]

**Additional file 4.** Definitions for Normalization Process Theory Core Mechanisms and Sub-Components.

| **Coherence**  Sensemaking work people do to understand individually and collectively what they need to do to enact a new innovation. |
| --- |
| **Differentiation:** The sensemaking work people do to understand how a new innovation is different from current practices. |
| **Individual specification:** The sensemaking work individuals do to understand what they must do to utilize a new innovation and embed it into existing workflows. |
| **Communal specification:** The sensemaking work that people do collectively to create a shared understanding of the benefits of using a new innovation. |
| **Internalization:** The sensemaking work that people do to come to see value in using a new innovation. |
| **Cognitive Participation**  Relational work people do to create a community of practice committed to using a new innovation. |
| **Initiation:** Ensuring key people are working to move the introduction of a new innovation forward. |
| **Enrolment:** The work people do to organize/reorganize themselves and others to contribute to the work involved in new practices. |
| **Legitimation:** The work of ensuring that people believe it is right for them to be involved with the innovation and that they can make a valid contribution to it. |
| **Activation:** Once the work is underway, people define actions/procedures to sustain a change in practice and to stay involved with a new innovation. |
| **Collective Action**  Operational work to enact a new way of working with a new innovation. |
| **Interactional workability:** The work people do with each other to operationalize a new innovation. |
| **Skill set workability:** Fit between the allocation of work and existing skill sets to carry out the work needed to use a new innovation. |
| **Relational integration:** The fit between a new innovation and existing knowledge and confidence surrounding the innovation among groups and professions. |
| **Contextual integration:** Resource work to support the integration of a new innovation – i.e. morale, time, money, leadership. |
| **Reflexive Monitoring**  Evaluation work to understand how a new innovation impacts oneself and others. |
| **Systematization:** People working together to evaluate the effectiveness of a new innovation – involves the work of collecting information. |
| **Individual appraisal:** Individual appraisal of the work to use a new innovation and how it impacts themselves and the other work they do. |
| **Communal appraisal:** People work together to evaluate the value of a set of practices to utilize a new innovation. |
| **Reconfiguration:** Evaluation work among individuals or groups may result in changes to the way a new innovation is used. |

The definitions were adapted from May C, Finch T. Implementing, embedding, and integrating practices: an outline of normalization process theory. *Sociology*. 2009;43(3):535-554. One of the original authors is also an author of the current paper
